# Microglia are not required for prion-induced retinal photoreceptor degeneration

**DOI:** 10.1186/s40478-019-0702-x

**Published:** 2019-03-25

**Authors:** James F. Striebel, Brent Race, Katie Williams, James A. Carroll, Mikael Klingeborn, Bruce Chesebro

**Affiliations:** 1 Laboratory of Persistent Viral Diseases, Rocky Mountain Laboratories, National Institute of Allergy and Infectious Diseases, National Institutes of Health, 903 South Fourth Street, Hamilton, MT 59840 USA; 2 Department of Ophthalmology, Duke Eye Center, Duke University, Durham, NC 27710 USA

**Keywords:** Retina, Degeneration, Photoreceptors, Gliosis, Microglia, Macrophages, Müller cells, Prions, Prion protein, PLX5622, Scrapie, Cx3cr1 knockout

## Abstract

Degeneration of photoreceptors in the retina is a major cause of blindness in humans. Often retinal degeneration is due to inheritance of mutations in genes important in photoreceptor (PR) function, but can also be induced by other events including retinal trauma, microvascular disease, virus infection or prion infection. The onset of apoptosis and degeneration of PR neurons correlates with invasion of the PR cellular areas by microglia or monocytes, suggesting a causal role for these cells in pathogenesis of PR degenerative disease. To study the role of microglia in prion-induced retinal disease, we fed prion-infected mice a CSF-1 receptor blocking drug, PLX5622, to eliminate microglia in vivo, and the effects on retinal degeneration were analyzed over time. In mice not receiving drug, the main inflammatory cells invading the degenerating PR areas were microglia, not monocytes. Administration of PLX5622 was highly effective at ablating microglia in retina. However, lack of microglia during prion infection did not prevent degeneration of PR cells. Therefore, microglia were not required for the PR damage process during prion infection. Indeed, mice lacking microglia had slightly faster onset of PR damage. Similar results were seen in C57BL/10 mice and transgenic mice expressing GFP or RFP on microglia and monocytes, respectively. These results were supported by experiments using prion-infected Cx3cr1 knockout mice without PLX5622 treatment, where microglial expansion in retina was delayed, but PR degeneration was not. Contrary to predictions, microglia were not a causative factor in retinal damage by prion infection. Instead, newly generated PrPSc accumulated around the inner segment region of the PR cells and appeared to correlate with initiation of the pathogenic process in the absence of microglia.

## Introduction

Prion diseases are transmissible neurodegenerative diseases of the CNS which occur in humans and animals. These diseases are caused by an infectious agent composed primarily of a misfolded host-derived protein known as prion protein (PrP or PrPC). Hallmarks of prion disease in the CNS are deposition of misfolded protease-resistant prion protein (PrPres or PrPSc), as well as microgliosis, astrogliosis, and vacuolation of gray matter followed by neurodegeneration [3, 20]. Typically, gliosis occurs prior to vacuolation or neuronal death, suggesting that activated glia might be involved both in causing the pathogenesis and in participating in the host response against the infection and damage. Although prion disease primarily involves brain and spinal cord, other tissues such as peripheral nerves, retina, intestines and lymphoid tissues are also infected.

In the current paper, we studied retinal degeneration caused by prion infection, which has been known for many years [1, 5]. Due to the devastating nature of the brain degeneration seen in typical human prion diseases, retinal damage is not considered a major clinical issue. However, the unique spatial organization of neurons and other cells in retina provides an opportunity to study prion-induced neuropathogenesis in a slightly less complex context than brain, perhaps allowing more accurate understanding of the detailed mechanisms of neuronal pathogenesis.

Destruction of photoreceptors and loss of photoreceptor (PR) cell bodies is a major feature of the retinal pathology induced by infection with both human and animal prions [5, 15, 17, 19]. High levels of prion infectivity can be detected in retinal tissue of animals by in vivo bioassay [6, 18, 22]. In humans with prion disease, infectivity could be detected in ocular tissue [2], and prion seeding activity has been detected by RT-QUIC in retinas from CJD patients [30]. In multiple models, disease-associated PrPSc, astrogliosis, and microgliosis were observed in areas of degeneration in the PR areas [4, 17, 19, 22, 23, 39], thus mirroring the typical pathogenic findings in prion-infected brain.

The pathology of prion-induced retinopathy is similar to that seen in inherited PR degeneration diseases in humans. These diseases, known collectively as retinitis pigmentosa, are associated with over 44 different mutations in a variety of genes [40]. Inherited PR degeneration is usually associated with an influx of activated microglia into the PR areas, including the outer segment (OS), inner segment (IS) and outer nuclear layer (ONL). Many observations suggest that these microglia contribute to damage of PR cells [40–42]. For example, microglia have been suspected of contributing to PR degeneration by secretion of chemotactic and inflammatory cytokines, generation of reactive oxygen and nitrogen species, and by aberrant phagocytosis of living PR cells [35, 40]. Because of the evidence suggesting that microglia contribute to inherited PR degeneration, coupled with the similarities in pathology between these inherited diseases and prion-induced PR degeneration, we suspected that microglia also have a causative role in prion-induced retinal disease. To determine more precisely the role of microglia in various pathologic conditions, ablation of microglia has been attempted by several methods including administration of toxins or drugs targeted to microglia or use of transgenic mice to express toxins in microglia [38]. However, most of these methods have some technical drawbacks limiting their utility. Recently PLX5622, a new orally administered drug, which blocks the microglial CSF-1 receptor was found to rapidly eliminate most CNS microglia in vivo and has been beneficial in elucidating the role of microglia in a variety of systems [9–11, 37].

In our previous work using PLX5622 to study prion diseases, microglia could be suppressed for many months with continued drug administration [7]. Furthermore, in this study, ablation of microglia by treatment with PLX5622 revealed that microglia were not required for induction of clinical prion brain disease and instead had an important beneficial effect in delaying progression of prion brain disease by 20 to 30 days.

In the present paper, we used PLX5622 to study the role of microglia in prion-induced retinal degeneration. Due to the above-mentioned similarities between inherited PR disease and prion-induced PR disease, we hypothesized that microglia might be required for prion-induced retinal disease and might not have a beneficial effect as was seen in our earlier brain experiments. However, the present experiments using microglia ablation by PLX5622 did not support this hypothesis. The results indicated that after prion infection of retina, microglia were not required for PR degeneration. Moreover, there was evidence suggesting that PR degeneration in the absence of microglia was more rapid than when microglia were present. Thus, the contribution of microglia in brain and retina appeared to be similar during prion infection.

## Methods

### Ethics statement

All mice were housed at the Rocky Mountain Laboratories (RML) in an AAALAC-accredited facility in compliance with guidelines provided by the Guide for the Care and Use of Laboratory Animals (Institute for Laboratory Animal Research Council). Experimentation followed RML Animal Care and Use Committee approved protocol 2016–042.

### Mice

C57BL/10SnJ mice were obtained from an in-house breeding colony, these mice are noted as C57BL/10 throughout the paper. TgGFP/RFP mice were generated by crossbreeding Cx3cr1 knockout mice homozygous for the Cx3cr1-GFP targeted mutation (B6.129P-*Cx3cr1*^*tm1Litt*^/J,The Jackson Laboratory, Stock No: 005582) with Ccr2 knockout mice homozygous for the Ccr2-RFP targeted mutation (B6.129(Cg)-*Ccr2*^*tm2.1Ifc*^/J,The Jackson Laboratory, Stock No:017586) [21, 34]. Resultant offspring had heterozygous expression of both Cx3cr1 and Ccr2. In addition, green fluorescent protein (GFP) was expressed under promoter for Cx3cr1, prominently expressed by microglia in the CNS and red fluorescent protein (RFP) was expressed under the promoter for Ccr2, prominently expressed by monocytes [33]. All mice were group housed in transparent cages in a 12 h light (250-300 lx) /12 h dark cycle and food and water were available ad libitium.

### Scrapie inoculations

Mice (4–6 weeks old) were injected intracerebrally (i.c.) in the left hemisphere with 30 μl of a 1% (wt/vol) dilution of brain homogenate pools from C57BL mice terminally ill from 79A scrapie. Titer was determined in previous i.c. endpoint titration experiments and was 9.6 × 10^7^ LD_50_ (units = 50% infectious dose (ID_50_)/gm of brain) [36]. Therefore, mice received 2.9 × 10^4^ LD_50_ in a volume of 30 μl. Brain homogenates were diluted for inoculation in phosphate-buffered balanced saline (PBBS) pH 7.2, supplemented with 2% fetal bovine serum (Hyclone, Logan, UT). Observations were made daily to assess signs of scrapie disease, which included ataxia, altered gait, wasting, kyphosis, hind limb weakness, somnolence and immobility. Mice were judged clinical when they displayed severe, consistent neurologic signs. At selected time points, both pre-clinical and clinical, mice were euthanized by isoflurane anesthesia overdose followed by perfusion with 10 ml of saline. Eyes were collected for use in immunohistochemistry or whole retinal flat mount preparations.

### PLX5622 treatment

To deplete retinal microglia, mice (C57BL/10 and tgGFP/RFP) were fed purified rodent diet AIN-76A (D10001, Research Diets, Inc.) with or without supplementation with compound PLX5622 (1200 mg/kg chow, kindly provided by Plexxikon Inc., Berkeley, CA). Specifically, C57BL/10 mice were fed control chow for 14 days after 79A scrapie-inoculation to allow mice to convalesce. Then “treated” mice were switched to PLX5622 supplemented chow and maintained on this diet until the experimental endpoint. TgGFP/RFP mice received a delayed treatment protocol; after inoculation with 79A scrapie, mice were fed control chow for 90 days, and then the “treated” group was switched to chow supplemented with PLX5622. Mice were then maintained on supplemented chow until the experimental endpoint.

### Histology and Immunohistochemical detection of PrPSc, GFAP, Iba1, GFP and RFP

Eyes for immunohistochemistry were removed, placed in 10% neutral buffered formalin for 3 to 5 days and then processed by dehydration and embedding in paraffin. Each eye was embedded as a single block and 5 μm sections were cut using a standard Leica microtome, placed on positively charged glass slides, and air-dried overnight at room temperature. The following day slides were heated in an oven at 60 °C for 20 min. A Ventana automated Discovery XT stainer was used for deparaffinization, antigen retrieval and staining, unless noted otherwise below.

PrP antigens were exposed by incubation in CC1 buffer (Ventana) containing Tris-Borate-EDTA, pH 8.0 for 100 min at 95 °C. Staining for PrP was done using human anti-PrP monoclonal antibody D13 [28] which was obtained from tissue culture supernatants made in our laboratory from CHO cells expressing the D13 antibody construct, which were kindly provided by Dr. R. Anthony Williamson, The Scripps Research Institute, La Jolla, CA. D13 culture fluid was used at a dilution of 1:100 for 2 h at 37 °C. The secondary antibody was biotinylated goat anti-human IgG at 1:500 dilution (Jackson ImmunoResearch, West Grove, PA.), and avidin-horseradish peroxidase was used with DAB as chromogen (DAB Map kit; Ventana Medical Systems, Tucson, AZ.).

Antigen retrieval for other targets was done using CC1 buffer at 100 °C for 44 min (Iba1), 60 min (RFP) or 20 min (GFAP). For GFP antigen retrieval, a Biocare Medical DC2002 Decloaking chamber with sodium citrate buffer at pH 6.0(0.01 M) was used for 20 min at 120 °C / 20 PSI and cooled to 80 °C. Microglia were stained with rabbit anti-Iba1 (1:2000) which was a gift from John Portis, Rocky Mountain Laboratories, Hamilton, MT. Retinal astrocytes and Müller cells were stained with rabbit anti-glial fibrillary acidic protein (GFAP) at (1:3500) (Dako). Green fluorescent protein (GFP) was stained using anti-GFP mouse monoclonal (Roche, ref.#11814460001, 1:200), and red fluorescent protein (RFP) was stained with anti-RFP rabbit polyclonal (ab124754, Abcam, 1:100). Primary antibodies were diluted in PBS with 1% normal goat serum and 0.1% Triton X-100. Diluent without antibody was used as a negative control. Ventana streptavidin-alkaline phosphatase protocol was used to detect Iba1 and GFAP as described previously [23] with the exception that Fast-Red chromogen was used. Detection of GFP and RFP also used the Ventana streptavidin-alkaline phosphatase protocol with secondary antibodies; biotinylated horse anti-mouse IgG (vector ba-2000) and biotinylated goat anti rabbit-IgG (Biogenex pre-dilute cat#HK3369R), respectively and DAB as chromogen. Slides were examined, and photomicrographs were taken and observed using an Olympus BX51 microscope and Microsuite FIVE software.

### Flat mount preparation and confocal microscopy

After enucleation, tgGFP/RFP mouse eyes were placed in 10% neutral buffered formalin for 2 h. Formalin was removed, and eyes were washed with PBS. The eyes were transferred to a culture dish and under a dissection microscope, the anterior part of the eye was removed with a circular cut at the *ora serrata*. The lens, iris and optic nerve were removed and using micro-forceps, light pressure was applied to the posterior area of the sclera until the retina separated from the retinal pigment epithelium (RPE) and sclera. Once the retina was carefully removed, it was flattened, vitreal side up on a microscope slide. To avoid curling, radial cuts were made in a symmetrical four-leaf pattern. ProLong Gold (ThermoFisher) mounting media was applied and the retina was coverslipped for viewing. Analysis of retinal flat mounts was completed on a confocal laser-scanning microscope (Zeiss LSM 710, Carl Zeiss, Germany). A Z-stack of 40–70, 1 μm optical sections was taken for each retinal area photographed, and data were analyzed using IMARIS software (version 8.4.1).

The number of mice studied by retinal flat mounts was as follows: ND group, 3 uninfected, 12 infected; PLX group, 2 uninfected, 10 infected. At dpi = 82 (ND only), 104, 118, 129 (PLX only), 131, 134 (PLX only), 144 (ND only), and 153 (ND only), retinas from 1 to 3 mice of each group were examined.

### Slide scanning,quantitative analysis of pathology and statistical analysis

Aperio Digital Pathology Systems (Aperio Technologies, Vista, CA) was used to scan, capture, and store whole slide images from histology samples. Immunohistochemical slides were scanned with the ScanScope® XT at 20X magnification. ImageScope™ software (Aperio ImageScope v11.1.2.760) and Aperio software (eSlide Manager Version 12.1.0.5029) was used to view, export and analyze e-slide images in JPEG format. Quantitative analysis of outer nuclear layer (ONL) thickness was performed on H&E-stained, 5 μm vertical sections showing complete retina at least 250 μm from the optic disc. To document disease-associated thinning of the ONL, three measurements, from the outer limiting membrane to the outer plexiform layer, were taken per mouse and averaged. The number of Iba1-positive, GFP or RFP cells in the photoreceptor layer was determined by counting the number of positive cells with nuclei in the IS and OS for two complete retinal sections. These numbers were averaged for each mouse. All statistical analysis was performed using GraphPad Prism software, version 7.04, details of statistical tests performed are described in the figure legends.

### TUNEL staining

Deparaffinized sections (prepared as described above) were stained using a Click-iT TUNEL Alexa Fluor 488 Imaging assay kit (Life Technologies, #C10245) following the included protocol. Stained sections were analyzed using an Olympus BX51 fluorescent microscope and Microsuite FIVE software. In order to quantitate apoptosis, the number of TUNEL stained nuclei were counted in two complete retinal sections, per mouse eye and averaged.

## Results

### Prion-induced retinal degeneration associated with gliosis and destruction of photoreceptor cells

In the present experiments, we studied the retinal degeneration process in mice after i.c. infection with scrapie strain 79A. In most experiments, we used double transgenic mice (tgGFP/RFP) in which microglia expressed GFP under control of the Cx3cr1 promoter and monocytes expressed RFP under control of the Ccr2 promoter, in order to be able to better distinguish microglia from monocytes [21, 34]. In some experiments C57BL/10 mice were also studied. After i.c. scrapie injection, prions infect the brain locally and rapidly spread via neuronal circuitry to retina as well as to various brain regions [17, 24]. In C57BL/10 mice and tgGFP/RFP mice, early clinical neurological signs such as abnormal gait and ataxia were noted starting as early as 125 dpi, and mice reached the clinical disease endpoint requiring euthanasia between 153 and 168 dpi.

**Fig. 1:**
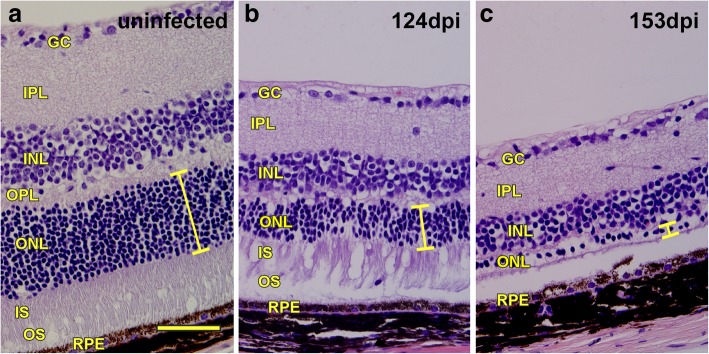
Progressive retinal degeneration in tgGFP/RFP mice after intracerebral 79A scrapie inoculation. **a** H&E stain of retina from a mock-inoculated mouse at 162 dpi showing normal retinal structure. Yellow bar indicates thickness of outer nuclear layer (ONL), which contains nuclei of photoreceptor (PR) cells. **b** Retina from a 79A scrapie-infected mouse at 124 dpi showing thinning of ONL (yellow bar), alteration of the IS and OS regions and presence of an infiltrating mononuclear cell in the OS region (arrow). **c** Retina from a 79A scrapie-infected mouse at 153 dpi shows nearly complete degeneration of ONL (yellow bar) and loss of IS and OS regions. Thinning of inner nuclear layer (INL) and inner plexiform layer (IPL) is also apparent. GC = ganglion cell layer, IPL = inner plexiform layer, INL = inner nuclear layer, OPL = outer plexiform layer, ONL = outer nuclear layer with photoreceptor cell nuclei, IS = inner segment of photoreceptors, OS = outer segment of the photoreceptors, RPE = retinal pigment epithelium. Scale bar = 100um

To follow the onset and progression of eye disease mice were euthanized at various times, and brain and eye tissues were examined by histopathology. In both C57BL/10 and tgGFP/RFP mice, signs of retinal damage were initially noted at 118 to 128 dpi when thinning of the outer nuclear layer (ONL) was first observed, photoreceptors appeared to be disorganized, and mononuclear cells were seen in the PR areas. At 150 to 165 dpi, there was extensive retinal degeneration as indicated by loss of photoreceptor cells and their nuclei in the ONL, examples of retinas from prion-infected tgGFP/RFP mice at 124 and 153 dpi are shown in Fig. 1.

**Fig. 2:**
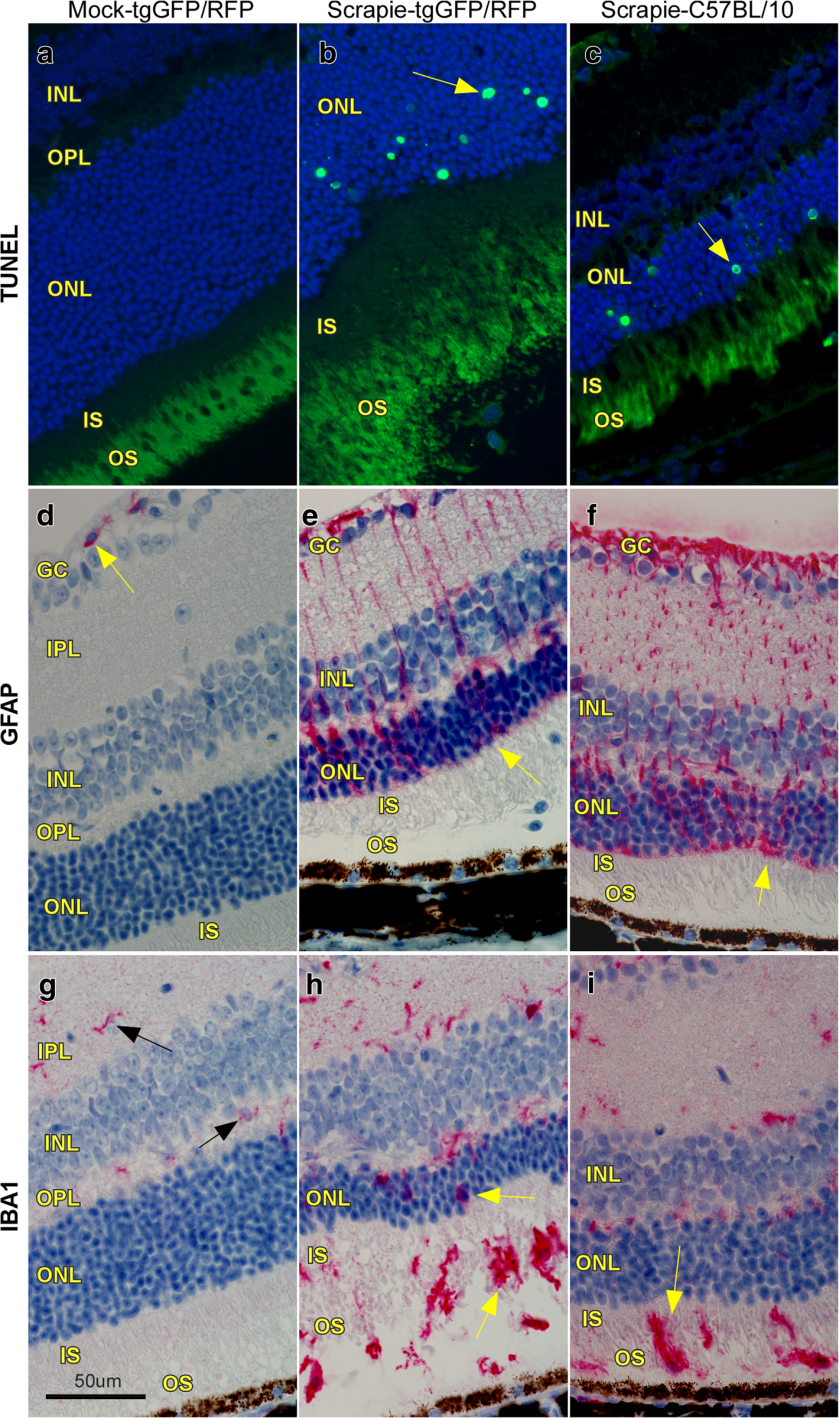
Histopathological changes in 79A scrapie-infected retinas. **a** TUNEL staining of mock-infected mouse shows no evidence of apoptosis. **b** Scrapie-infected tgGFP/RFP mouse retina with TUNEL-positive nuclei (arrow) in the ONL indicating active apoptosis at 124 dpi. **c** Scrapie-infected C57BL/10 retina showing TUNEL-positive nuclei in the ONL (arrow) at 144 dpi. **d** Anti-GFAP staining of mock-infected retina is restricted to perivascular astrocytes (arrow) in the GC layer. **e** & **f** In tgGFP/RFP and C57BL/10 scrapie-infected retinas, GFAP-positive (red) processes of activated Müller cells (astrocytes) extend from GC layer to outer limiting membrane (arrow) of the ONL. **g** Anti-Iba1 staining of mock-infected retina shows a few Iba1-positive microglia (arrows) in the IPL and OPL. **h** & **i** In both tgGFP/RFP and C57BL/10 scrapie-infected retinas, Iba1-positive microglia are now also in ONL, OS and IS regions (arrows). These cells have enlarged cell bodies and thick processes typical of activated microglia. Scale bar = 50 μm, each column contains images from one representative mouse

In our previous work on retinal degeneration induced by hamster scrapie prions, we found evidence for apoptosis as the mechanism of destruction of photoreceptor cells [22]. Similarly, in the present studies, using scrapie strain 79A to infect tgGFP/RFP and C57BL/10 mice, apoptosis was noted by TUNEL assay in the photoreceptor cell nuclei located in the ONL (Fig. 2a-c). Furthermore, staining with anti-GFAP showed presence of activated astroglia (Müller cells) with bipolar processes often spanning the width of the retina (Fig. 2d-f), and staining with anti-Iba1 showed many positive cells with the morphology of activated microglial cells. This was seen in all retinal layers, but most strikingly in the PR (ONL,OS and IS) areas (Fig. 2h-i), where Iba1-positive cells were rarely seen in normal animals (Fig. 2g).

### Depletion of microglia in retinas of uninfected mice after treatment with PLX5622

**Fig. 3:**
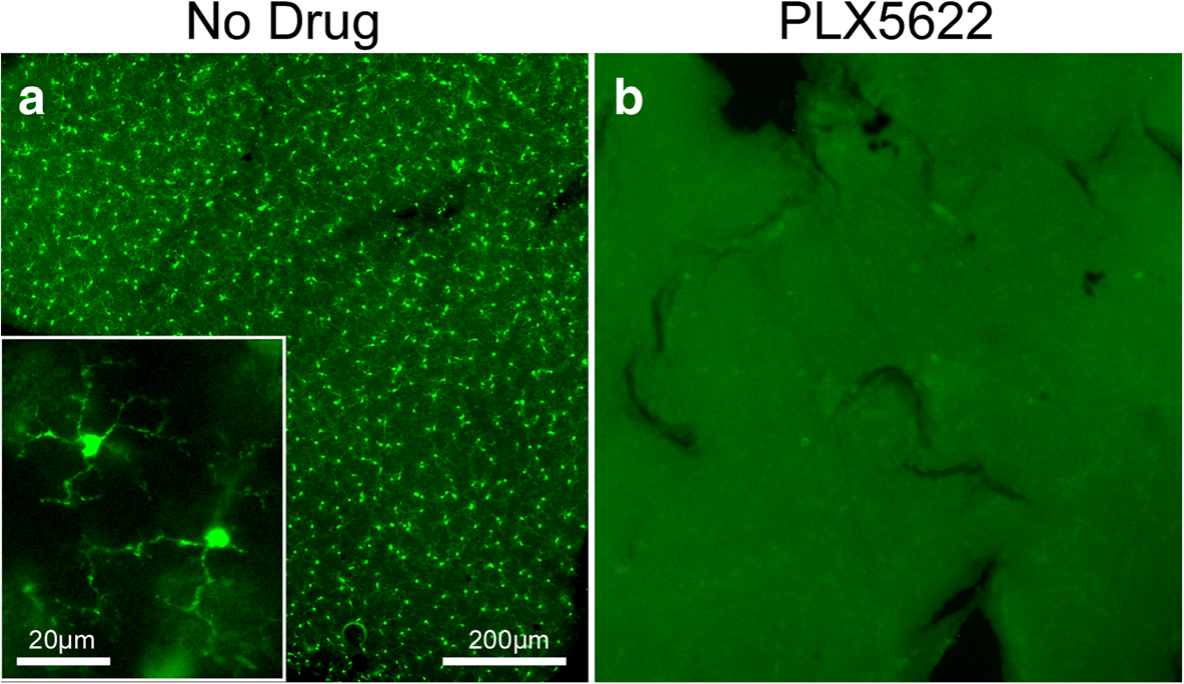
Ablation of retinal microglia following PLX5622 treatment. Retinal flat mounts from uninfected tgGFP/RFP mice without drug treatment (**a**) and with PLX5622 treatment (**b**). **a** In untreated mice, GFP-expressing microglia span the entire retina and appear highly branched with fine delicate processes (inset). **b** After 7 days of PLX5622 treatment, microglia have disappeared from the entire retina. Scale bars = 200 μm for a and b, 20 μm for inset

Although the preceding experiments detected microglia in retinas of scrapie-infected mice around the time of retinal degeneration, it was unclear whether these microglia were initiators of the damage or responders to the damage, or both. To investigate this matter, mice were given the CSF-1 receptor blocking drug, PLX5622, to ablate microglia to study their role in retinal degeneration. In uninfected tgGFP/RFP mice, oral administration of PLX5622 led to dramatic reduction of microglia in retina by 7 days post-treatment (Fig. 3a, b), and similar results were seen in retinas of C57BL/10 mice. This reduction was maintained as long as treatment was continued, in some cases over 180 days [7] .

### PLX5622 treatment of scrapie-infected C57BL/10 mice

To investigate the effect of microglia depletion during scrapie infection, scrapie-infected C57BL/10 mice were started on PLX5622-containing chow at 14 dpi, similar to previous experiments studying scrapie brain disease [7]. PLX5622-containing chow was maintained for the duration of the experiment (PLX group). Additional scrapie-infected mice received chow with no drug (ND group) to serve as a control group. In the ND group, early clinical signs were detectable around 125 dpi, and severe signs requiring euthanasia were seen between 155 and 165 dpi. In contrast, in the PLX group early clinical signs were noted around 105 dpi and severe signs were seen 129 and 139 dpi. Thus, as was reported previously [7], treatment with PLX5622 accelerated the clinical course of scrapie brain disease by about 20 days compared to untreated ND mice.

**Fig. 4:**
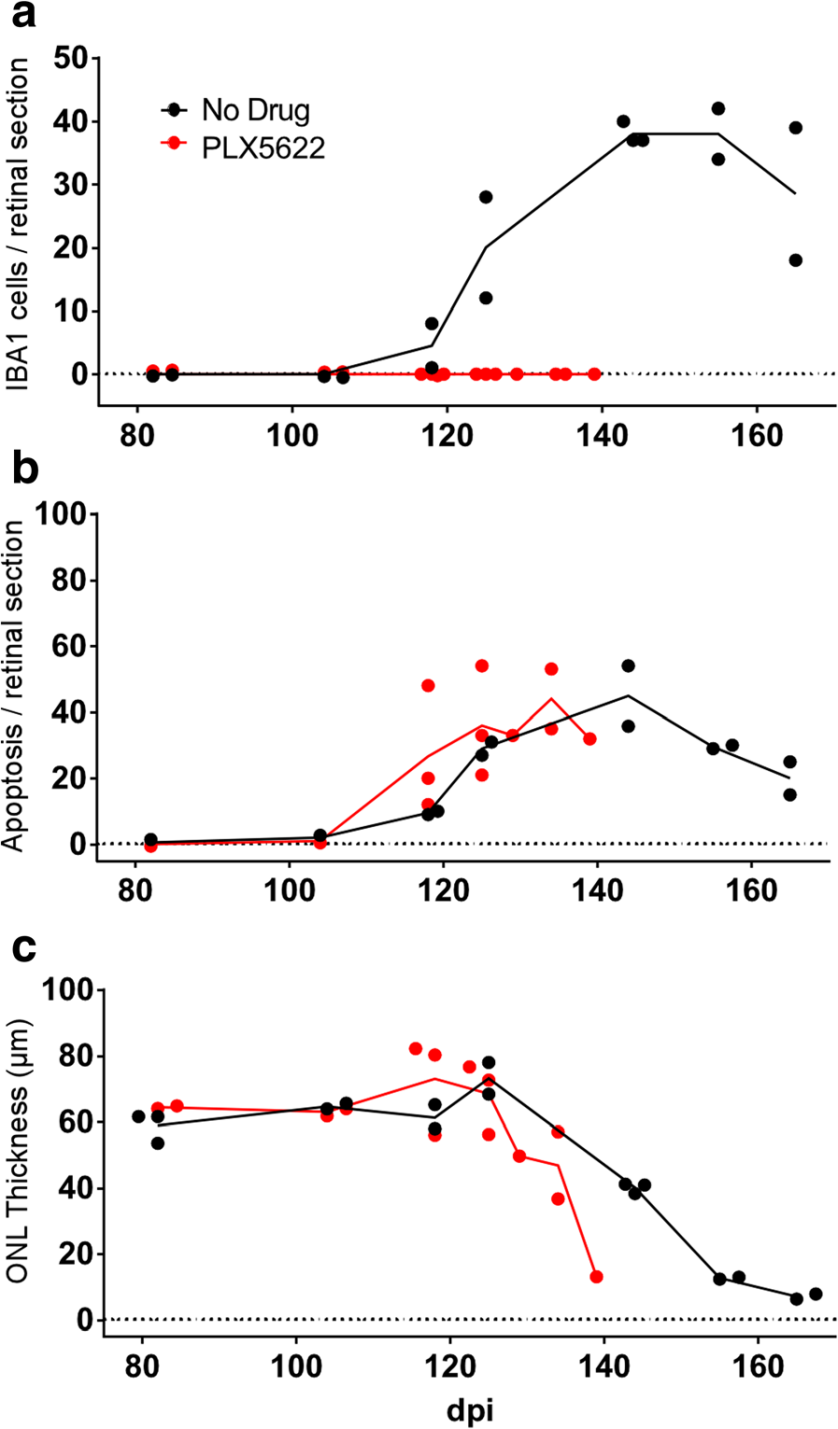
Scrapie-induced retinal degeneration in C57BL/10 mice in presence or absence of retinal Iba1-positive cells. On all graphs each dot denotes the mean value of counts from two sections of a single mouse, lines connect mean values at each timepoint, and lines end following euthanasia of final clinical mouse. PLX5622 treatment was initiated at 14dpi. **a** Number of infiltrating Iba1-positive cells in the photoreceptor layer increased in the no drug (ND) group from 124 to 162 dpi, whereas Iba1-positive cells were not observed in the PLX5622-treated group. **b** Apoptosis was detected by observation of TUNEL-positive cells in the outer nuclear layer (ONL) in both ND and PLX groups. Apoptosis was not dependent on presence of Iba1-positive cells. **c** ONL thickness (μm) decreased in both ND and PLX groups indicating that loss of photoreceptor nuclei in the ONL did not depend on Iba1-positive cells. ONL thinning in PLX group which lacked microglia appeared to be slightly faster than in the ND group. However, best-fit line slopes for points from ND and PLX groups between 118 and 164 dpi were not significantly different, *p* = 0.101

In the present experiments, retinas were removed at various times for histological examination. In C57BL/10 mice, in the ND group, Iba1-positive cells appeared in the IS and OS layers of the PR region starting at 125 dpi with a peak of around 30–40 cells per retinal section circumference between 140 and 160 dpi (Fig. 4a). However, in the PLX group, Iba1-positive cells were not present in any of the IHC sections analyzed. Therefore, PLX5622 appeared to abolish the host Iba1-positive cell response to scrapie infection in retina suggesting that the infiltrating cells might be mostly microglia.

As noted in our previous report [22] and above in Fig. 2a-c, apoptosis in the ONL region is an early feature of prion-induced retinal damage. Therefore, to investigate whether treatment of C57BL/10 mice with PLX5622 interfered with this damage, apoptosis was also studied in retinal tissue of scrapie-infected ND and PLX mice. In this experiment, similar levels of apoptosis were seen in the ONL of retinas in both ND and PLX groups starting at 118 dpi (Fig. 4b). Thus, scrapie-induced photoreceptor cell death appeared to proceed similarly in the presence or absence of Iba1-positive cells. Furthermore, progressive severe depletion of photoreceptor cells, as measured by thinning of the ONL occurred in both PLX and ND mice (Fig. 4c). There was a suggestion that ONL thinning appeared slightly earlier in the PLX group compared to the ND group; however, this difference was not statistically significant.

### PLX5622 treatment of scrapie-infected tgGFP/RFP mice

To distinguish between microglia and monocytes, which are both Iba1-positive, we did similar scrapie infection experiments using tgGFP/RFP mice, which express GFP in microglia and RFP in monocytes as described in methods. In addition, we were concerned that early treatment with PLX5622 might influence the spread of scrapie from the brain injection site to the eye, so we waited until 90 dpi before starting PLX5622 treatment, as this schedule was also found previously to be highly effective in ablating microglia in brain [7].

**Fig. 5:**
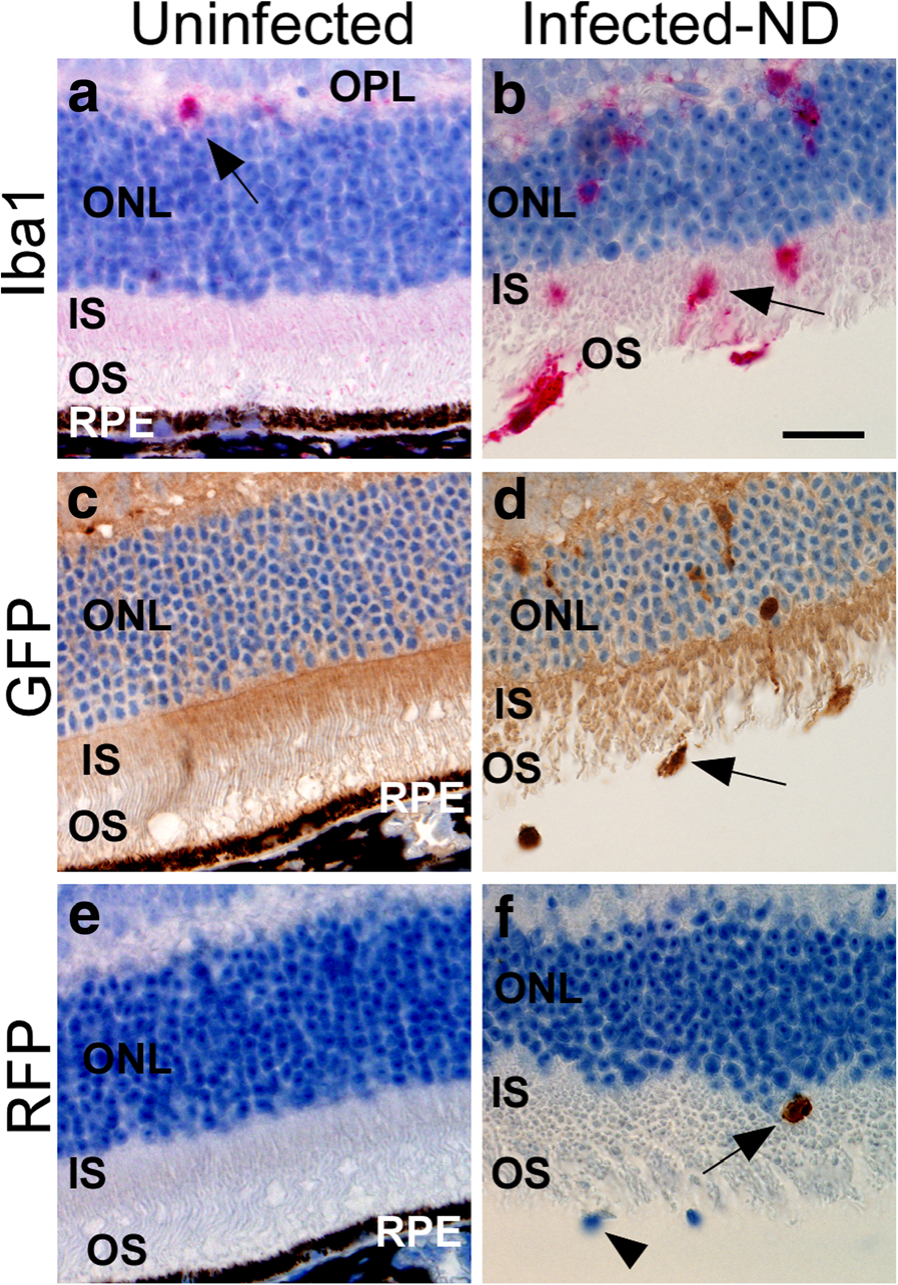
Immunohistochemical staining of tgGFP/RFP retinas using anti-Iba1, anti-GFP and anti-RFP antibodies. **a** anti-Iba1 staining of uninfected retina shows absence of microglia in the ONL, IS, and OS, one Iba1-positive cell (red) is visible in the OPL (arrow). **b** In response to scrapie infection of retina, there is significant migration of Iba1-positive cells (arrow) to the ONL, IS and OS. **c** anti-GFP staining (brown), indicative of Cx3cr1 expression in microglial cells, is not present in the ONL, IS and OS of the uninfected retina. **d** In the infected retina, microglia expressing GFP (brown) are present at a similar density to Iba1-positive cells in (**b**). **e** anti-RFP staining (brown), specific for Ccr2-expressing monocytes is not visible in the uninfected retina. **f** A rare RFP-positive cell (arrow) is present in the IS of a scrapie-infected retina. Note the two unstained nuclei (arrowhead), which are likely Iba1/GFP positive cells. Scale bar is 25 μm

**Fig. 6:**
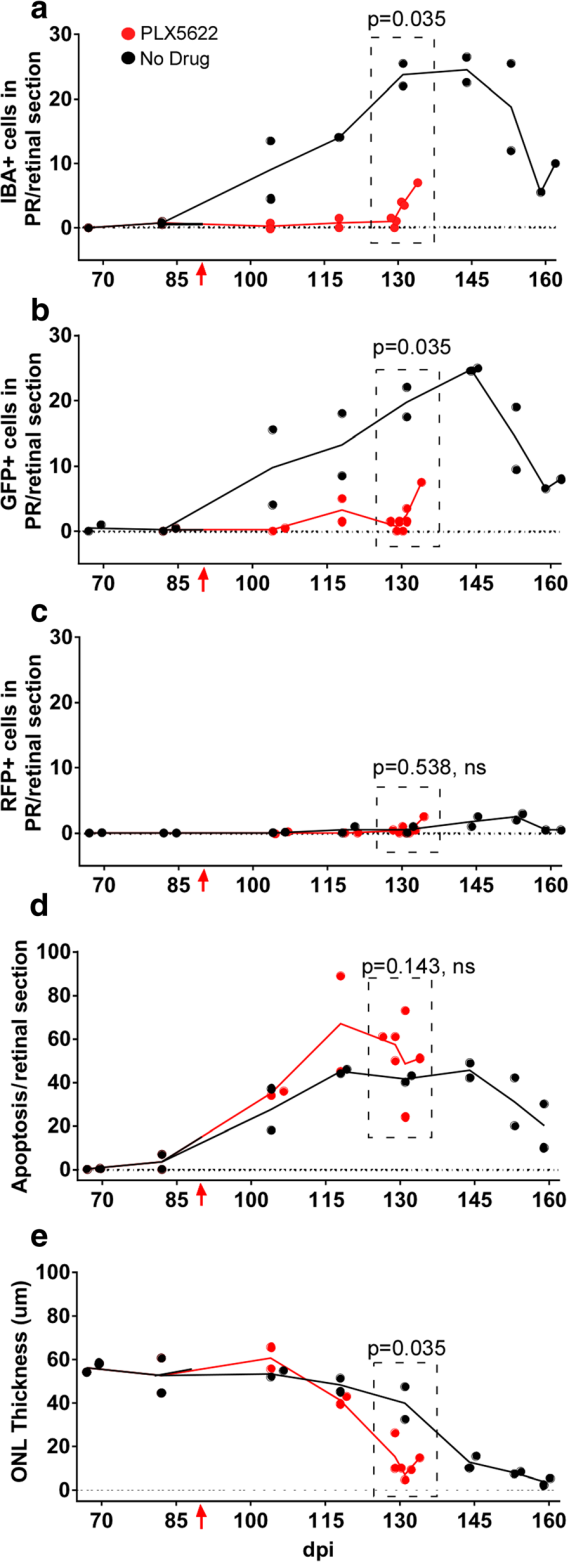
Scrapie-induced retinal degeneration in tgGFP/RFP mice in presence or absence of retinal microglia. PLX5622 treatment was initiated at 90 dpi (red arrow). **a** Number of Iba1-positive cells in the photoreceptor layer increased in the no drug (ND) group, but remained low in the PLX5622-treated group. **b** The GFP-positive cells invading the OS and IS regions were also counted, as they indicated Cx3cr1 expression and a microglial phenotype. These were elevated at similar timepoints as were seen for Iba1. **c** Late in disease only rare cells were stained with anti-RFP antibody, which was a marker of Ccr2 expression and a monocyte-derived macrophage phenotype. **d** Apoptosis was detected by TUNEL staining in both ND and PLX groups in the ONL. **e** ONL of both ND and PLX groups showed marked thinning starting at 128 dpi. Comparison of ND vs PLX points around the maximum ONL thinning (129-134dpi, dotted boxes), was done by one tailed Mann-Whitney test, and *p*-values are shown on each panel

In this experiment using TgGFP/RFP mice, we determined whether the Iba1-positive cells observed in retinal sections were microglia or monocytes by immunohistochemistry using anti-GFP or anti-RFP antibodies. Figure 5 shows examples of the IHC staining, where antibodies to Iba1, GFP and RFP were compared. In these studies, comparison of PLX versus ND groups in tgGFP/RFP mice gave very similar results to those seen previously using C57BL/10 mice. In the ND-group, Iba1-positive cells increased in the IS and OS layers of the PR region starting at 105 dpi with a peak between 118 and 145 dpi. In contrast, in the PLX group, rare Iba1-positive cells were detected in these same layers of the PR region at the latest time-points (Fig. 6a). Using IHC with anti-GFP and anti-RFP, large numbers of GFP-positive cells were detected in the retinal PR region of ND mice between 105 and 162 dpi, but numbers were much lower in the retinal PR region of PLX mice (Fig. 6b). In contrast, few RFP-positive cells were detected in either ND or PLX mice (Fig. 6c). Detailed data on all mice studied are shown in Table 1. Based on these data, we concluded that the detectable Iba1 cell response consisted mostly of microglia, and monocytes were only rarely detected. In addition, PLX5622 treatment abolished most of the microglial response in PLX retinas.

**Table 1:** The effect of PLX5622 treatment on the types of cells infiltrating the retinal inner and outer segments during scrapie disease

		Time (dpi)							
		82	104	118	129/131	134	144	153	159/163
No Drug	*n*	2	2	2	2	0	2	2^a^	2^a^
	*IBA1*	1,1^b^	5,14	15,14	26,22	*na*	23,27	26,12	6,10
	*GFP*	1,0	4,15	9,18	18,22	*na*	25,30	19,10	6,8
	*RFP*	0,0	0	0,2	1,0	*na*	1,3	3,2	1,1
PLX5622^c^	*n*	0	2	2	5	1^a^	0	0	0
	*IBA1*	*na*	0,0	0,2	1,2,2,4,4	7			
	*GFP*	*na*	1,0	1,2	0,2,2,4,2	8			
	*RFP*	*na*	0	0	1,1,0,1,0	3			

*dpi* Days post inoculation, *n* Number of animals tested, *IBA1* Ionized calcium binding adaptor molecule 1 (microglia, monocyte-macrophage marker), *GFP* Green fluorescent protein (microglial marker), *RFP* Red fluorescent protein (monocyte-macrophage marker), *na* not available

^a^euthanized for severe clinical disease

^b^each number represents the mean number of IHC marker-positive, nucleated cells counted in the inner or outer segment areas per two retinal sections from one mouse. Fractional numbers were rounded up to next whole integer

^c^PLX5622 treatment was initiated at 90dpi and the last PLX5622-treated animal was euthanized at 134dpi

In spite of the difference in numbers of Iba1-positive microglial cells in the two treatment groups, apoptosis in the ONL was similar in both groups suggesting ongoing retinal degeneration (Fig. 6d). In addition, this retinal apoptosis was associated with direct evidence of thinning and degeneration of the ONL in both ND and PLX groups (Fig. 6e). Here thinning appeared to be faster in the PLX group, and this difference was statistically significant (*P* = 0.035). These data supported two conclusions: (1) Prion-induced retinal degeneration did not require presence of microglia or other Iba1-positive cells, and (2) lack of microglia appeared to accelerate the retinal degeneration observed.

### Study of retinal flat mounts of ND and PLX mice by confocal microscopy

In order to obtain a 3-dimensional view of the ongoing degeneration process, intact retinas from infected ND and PLX tgGFP/RFP mice were gently fixed, and prepared as flat mounts for examination by confocal microscopy. In these preparations, expression of GFP and RFP was detected directly without the use of any antibodies. Data was gathered by obtaining 40 to 50 optical slices of approximately 1 μm thickness in selected areas.

**Fig. 7:**
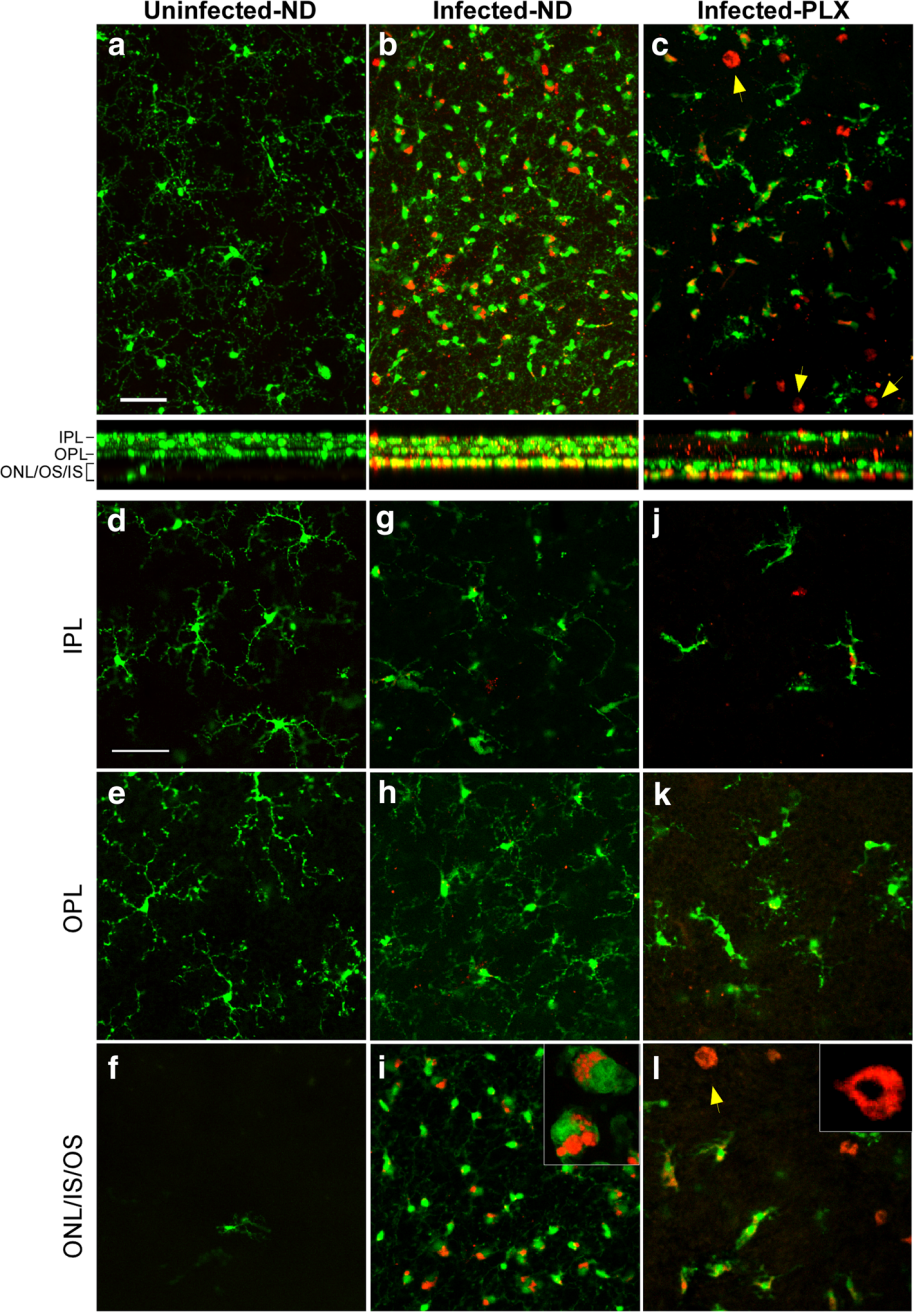
Retinal flat mount study of tgGFP/RFP mice by confocal microscopy. a,b,c. Z-stacks of 40–50 optical 1 μm sections of retinal flat mounts from tgGFP/RFP mice which express GFP (green) in microglia and RFP (red) in monocytes. **a** Uninfected mouse shows scattered green microglia with long delicate processes. Cross-section below with XZ dimension shows most green cells are in the OPL-IPL areas. **b** At 159dpi infected mouse from ND group has a large increase in green microglia with larger cell bodies and stubby processes typical of activated microglia. Some green/red dual stained cells can also be seen. XZ cross-section shows many of these cells are now in the ONL/IS/OS region. **c** At 134 dpi an infected mouse from the PLX group shows reduced number of green microglia and a few green/red cells as well. In addition, a few round all red cells, probably monocytes can be noted (arrows). **d-f** Single optical sections from uninfected mouse show that microglia are mostly in the IPL and OPL layers. **g-i** Optical sections of 159 dpi ND mouse show that green microglia in the IPL and OPL layers have processes similar to those seen in uninfected mice, whereas in ONL,IS,OS region green microglia and green/red microglia appear activated and have stubby processes. Some cells have red material apparently internalized in phagolysosomes (inset). **j**-**l**. Optical sections of 134 dpi PLX mouse also have a few green microglia with processes in IPL and OPL layers. In ONL/IS/OS region round cells with red cytoplasm appear to be monocytes (arrow). All scale bars = 50 μm. Number of mice studied at each time-point for each group is indicated in the methods section

Examination of the complete Z-stack of all 40–50 slices in the plane of the flat retina is shown for an uninfected mouse in Fig. 7a, where the GFP-expressing (green) microglia appeared to have small cell bodies and highly arborized processes typical of normal microglia. These cells were located mostly in the IPL and OPL regions as seen in the cross-section view of the stack. RFP-expressing (red) cells were very rare, and none were detected in this field.

In contrast, in the retina of a representative ND mouse at 159 dpi, microglia were much more abundant, and cell bodies were mostly large, and processes were short and stubby, consistent with activated microglia. Some cells had both green and red fluorescence, but the morphology of these cells was similar to the green-only microglia. In the cross-sectional view, many of these microglia were in the ONL/IS/OS region, similar to what was observed in the previously shown paraffin sections using IHC (Fig. 5b and d).

The retina of a representative PLX mouse from 134 dpi (Fig. 7c) showed a very low number of GFP-expressing microglia with an activated morphology, plus a few green/red cells also with microglial morphology. However, this retina also had several rounded red cells which appeared to be RFP-expressing monocytes. On the cross-sectional view, these cells were mostly located in the ONL/IS/OS regions.

**Fig. 8:**
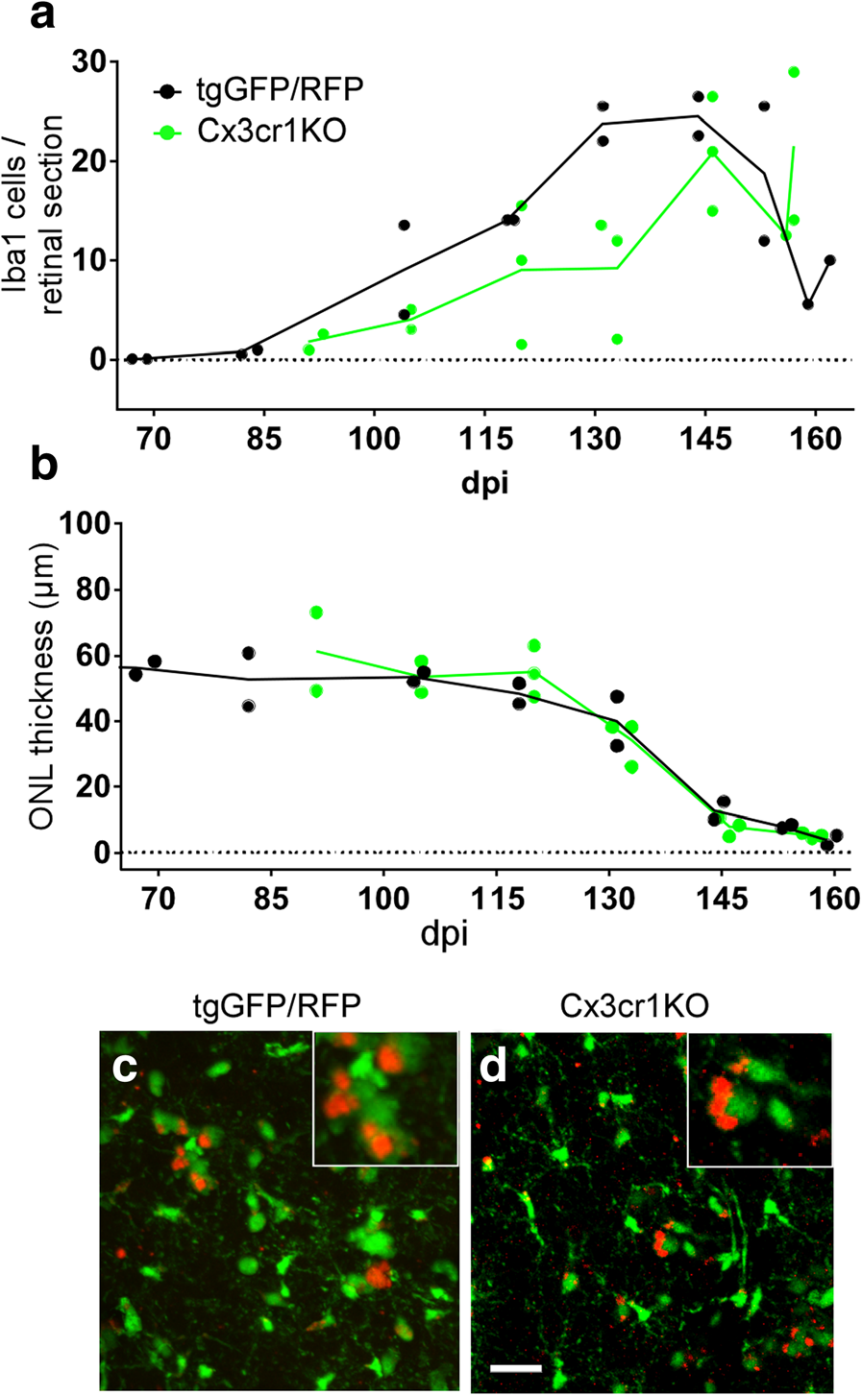
Comparison of microglial migration to photoreceptor area and PR thinning in scrapie-infected mice with and without expression of Cx3cr1. **a** After 79A scrapie infection, a delay in the number of Iba1-positive cells migrating to the PR areas was seen in Cx3cr1 knockout mice vs tgGFP/RFP (Cx3cr1 heterozygous) mice. Levels became similar in both mouse strains after 145dpi. **b** No difference was noted in the timing of ONL thinning in Cx3cr1 knockout mice vs tgGFP/RFP mice, suggesting that the delay of microglia had no effect on timing of ONL degeneration. **c and d** Retinal flat mounts examined by confocal microscopy show green microglia in both tgGFP/RFP mice (**c**) and Cx3cr1KO mice (**d**). Both mice also had red/green cells thought to be green microglia which had phagocytosed rhodopsin and/or other outer segment material, which was seen as red autofluorescence [25, 27]. Cx3cr1 mice do not express any RFP, so the red in these mice could not be RFP. Scale bar = 25 μm

For a more detailed view of the cell morphology, individual optical slices from each of these three mice were shown for three different levels. Figure 7d and e show normal microglia in the IPL and OPL regions with highly branched processes and small cell bodies. Rare microglia were seen in the ONL/IS/OS slice (Fig. 7f). In the ND mouse, a few microglia were detected in the IPL and OPL slices (Fig. 7g and h), but the majority of both the green and green/red microglia were in the ONL/IS/OS region (Fig. 7i). Here, the green/red cells appeared green over much of the cell body and the red appeared to be sequestered in cytoplasmic vacuolar structures resembling phagolysosomes. (see insert on Fig. 7i). Similar red fluorescent phagolysosomes were seen in prion-infected transgenic mice expressing GFP in microglia but lacking RFP expression, see Fig. 8c and d. This suggested that the red in these green/red cells was not RFP, but rather autofluorescence from phagocytosed outer segment material, as noted in other studies [25, 27].

In the PLX mouse, a few GFP expressing microglia were seen in the IPL and OPL regions (Fig. 7j and k), but in the ONL/IS/OS region fluorescent cells were either all red or mixed green/red (Fig. 7l). The round morphology of the all red cells was consistent with monocytes (Fig. 7l insert). However, the green/red cells were morphologically similar to activated microglia, which showed limited repopulation very late in disease [7].

In summary, microglial activation and invasion of the photoreceptors did not appear to be required for the process of prion-induced retinal degeneration. Monocytes were also likely not required, as they appeared late after the degeneration, consistent with a role in the clearance of the debris from dying cells.

### Prion-induced photoreceptor degeneration in Cx3cr1 knockout mice

As a further test of the role of microglia in prion-induced photoreceptor degeneration, we also studied prion-induced retinal disease in mice homozygous for expression of a GFP transgene in place of the Cx3cr1 open reading frame, resulting in knockout of Cx3cr1 expression (tgGFP-Cx3cr1KO) [8]. The Cx3cr1-Cx3cl1 axis, a neuron-to-microglia signaling chemokine axis, has been shown to influence microglial activation, migration and proliferation in several retinal disease models [35]. In our experiment, these mice showed a delay in recruitment of microglia to the IS and OS following prion infection compared to TgGFP/RFP mice (Fig. 8a). However, this reduction in infiltrating microglia did not reduce the rate of prion-induced ONL thinning (Fig. 8b), suggesting that microglia were not required for prion-induced retinal PR degeneration. Thus, these results support the data obtained from the previous PLX5622-induced microglial ablation experiments.

Comparison of retinal flat mounts from these mouse strains by confocal microscopy revealed both green microglia and green/red microglia-like cells in both mouse strains (Fig. 8c and d). Since the GFP-Cx3cr1KO mice did not express RFP, the red in these mice cannot be RFP (Fig. 8d). Rather, as other studies have noted, this red fluorescence is likely to be autofluorescence of phagocytosed outer segment material by microglia, similar to what was suggested previously in tgGFP/RFP mice (Fig. 7b and i) [25, 27].

### Detection of PrPSc in inner segment (IS) region of retinas at various times after scrapie infection

**Fig. 9:**
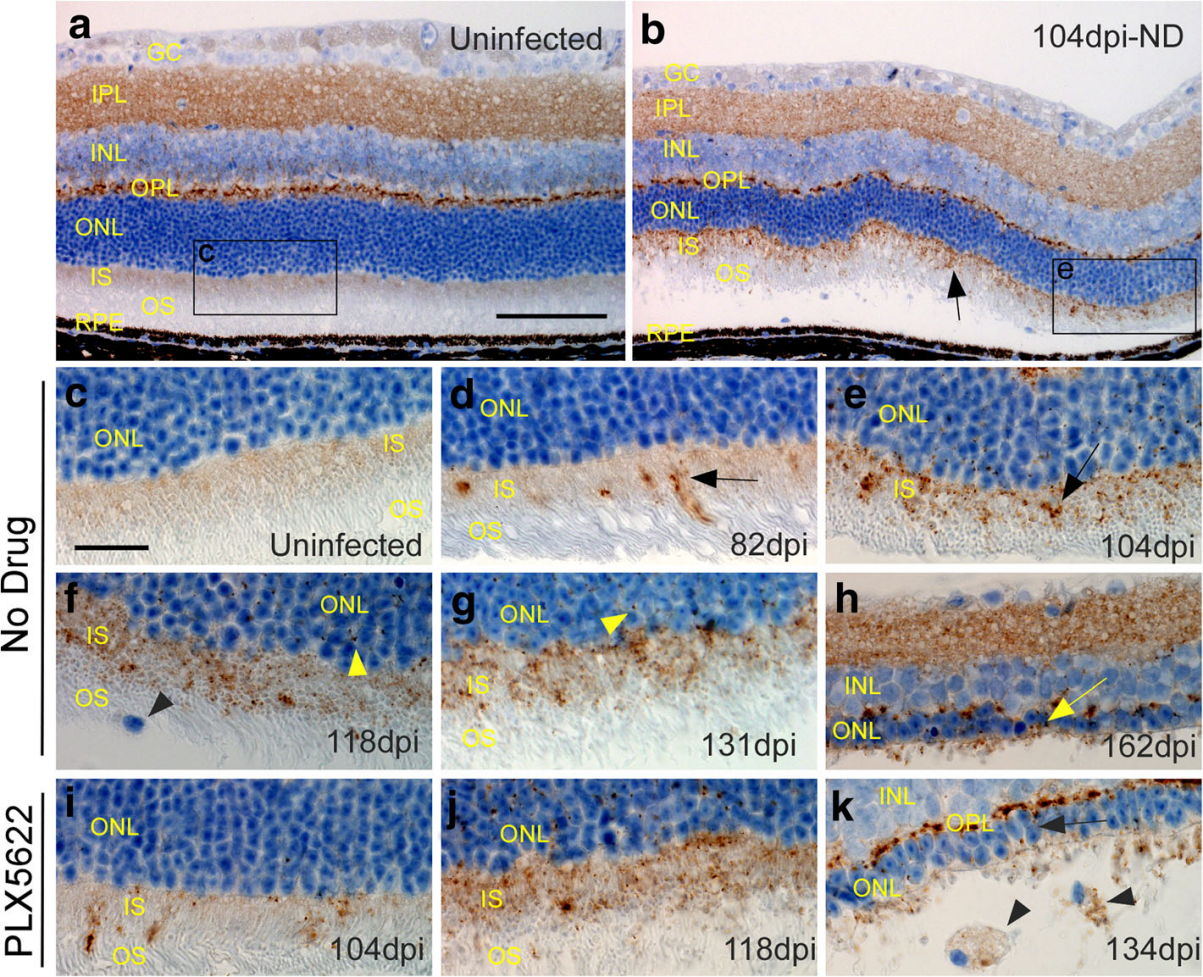
PrPSc deposition in retinas at various times after 79A scrapie infection. All panels show tgGFP/RFP mouse retinal sections stained with D13 anti-PrP monoclonal antibody. **a** In a control uninfected mouse, PrP staining (brown) was seen in the IPL, INL and OPL regions. In addition, a faint tan stain could be seen in the IS region of the photoreceptor cells. All the staining in this field was likely to be normal host PrPC since the mouse was uninfected. Furthermore, these areas were not stained in PrP null mice. **b** At 104 dpi in aninfected mouse from the ND group additional new PrP staining in punctate granular accumulations was observed in the IS region (arrow). This material appeared to be disease-associated PrPSc. **c-k.** More detailed view of the IS region was seen at higher magnification. **c** Uninfected mouse with faint brown blush in IS region. **d** Eighty two dpi ND mouse with earliest appearance of clumped PrPSc staining in IS (arrow). **e**, **f**, **g** On subsequent days (104, 118, 131dpi) PrPSc appeared to accumulate progressively in the IS region (arrows), and small punctate deposits could also be seen in the ONL (yellow arrowheads). One mononuclear microglial cell was present in the OS layer (black arrowhead). **h** At 162dpi severe retinal degeneration was observed, and the ONL was reduced to a single layer of nuclei (yellow arrow). OS and IS regions were almost totally absent, and only faint PrPSc staining remained. **i**, **j** In PLX5622-treated mice at 104 and 118dpi, PrPSc staining was similar to staining in the ND mice. **k** At 134dpi severe retinal degeneration was seen. Two mononuclear cells with phagocytosed PrPSc were seen in the area of the former OS region (black arrowheads). These pictures are representative of the following mice: 4 uninfected ND, 16 infected ND and 10 infected PLX treated. Mice were examined at 9 timepoints between 67 and 163 dpi, and representative mice from timepoints showing typical early to middle retinal degeneration were shown in this figure. a, b scale bar = 100 μm, c-k scale bar = 25 μm

Microgliosis, astrogliosis and deposition of disease-associated PrPSc are important hallmarks of prion disease in brain and spinal cord, and gliosis and PrPSc often co-localize with neuronal damage. To detect areas of PrPSc deposition, scrapie-infected tgGFP/RFP retinas from the PLX and ND groups were examined by IHC using anti-PrP monoclonal antibody D13. This antibody is reactive with both the normal cellular PrPC and the disease-associated PrPSc isoform. In control uninfected mice, PrP staining was seen in multiple retinal layers, including ganglion cell layer (GC), IPL, INL, and OPL, as well as faint staining in the inner segment (IS) of the PR region (Fig. 9a). Because these mice were not scrapie-infected, this staining was assumed to be PrPC, which is the normal isoform of PrP and is seen in many cell types and tissues. In a representative scrapie-infected ND mouse at 104 dpi, in addition to the PrPC staining seen in uninfected mice, PrP staining was detected in the IS region of the PR cells (arrow, Fig. 9b). This staining was likely to be PrPSc, as it was not seen in uninfected mice. A time course of the development of PrPSc detection in the IS region is shown at higher magnification (Fig. 9c-k and in Table 2, where all the mice studied are indicated). In ND mice, the earliest PrPSc detection was scattered areas of granular punctate staining in the IS region at 82 dpi (Fig. 9d). Subsequently, at 104, 118 and 131 dpi, more numerous and sometimes confluent areas of staining were found in the IS region and also in scattered punctate spots in the ONL (Fig. 9e, f, and g). At 162 dpi near the clinical endpoint in ND mice, the OS and IS layers were mostly gone and the ONL layer was reduced to a thickness of 1–2 nuclei (Fig. 9h). PrP staining at this time was mostly in the IPL and OPL, and PrPSc could not be distinguished from PrPC.

**Table 2:** The effect of PLX5622 drug treatment on PrPSc deposition in 79A scrapie-infected retinal inner segment, as detected by anti-PrP (D13) antibody staining

	Time (dpi)								
	67	82	104	118	129/131	134	144	153	159/163
No Drug	0,0^a^	1,1	2,2	2,2	3,3	*na*	D,3	D,D	D,D
PLX5622^b^	*na*	*na*	2,2	2,2	3,3,3,D,D	D	*na*	*na*	*na*

*dpi* Days post inoculation, *na* Not available

^a^Each number represents mean PrPSc score of two sections from one mouse. Entire retinal sections were scored for PrPSc present in the IS, as follows; *0* = No staining visible in the inner segment over entire retinal section; *1* = Low: less than 50 scattered deposits; *2* = Medium: more than 50 deposits; *3* = High: staining confluent along entire section; D, degenerated inner and outer segment, so assessment of staining not possible

^b^PLX5622 drug treatment was initiated at 90dpi, thus mice were not available at 67, 82 dpi. Last PLX5622-treated animal was euthanized at 134dpi

In the PLX-treated group, PrPSc was detected at 104 and 118 dpi in the IS region and in the ONL and was similar to the findings in the ND group (Fig. 9i and j). There was no suggestion that PrPSc was deposited earlier or in higher amounts in PLX-treated mice versus ND mice (Table 2). Thus, absence of microglia did not appear to alter levels or pattern of PrPSc deposition in retina. At the clinical end point of 134 dpi in the PLX group, retinal degeneration was advanced, no distinct PR layer could be seen, and the ONL had a thickness of only 2–3 nuclei (Fig. 9k).

In conclusion, retinal PrPSc could be detected at increasing levels in the IS region of the photoreceptor cells. However, there was no definitive difference in the time course of appearance of this PrPSc in ND versus PLX mouse groups (Fig. 9d-k and Table 2).

## Discussion

In many types of photoreceptor degeneration, activated microglia have been detected near sites of retinal damage [32, 35]. Since microglial responses often involve secretion of proinflammatory cytokines and production of nitric oxide and reactive oxygen species, microglia have been thought to be an important causative factor in PR damage. In several experimental models, when microglia were genetically inhibited or blocked with drugs, pathology was decreased, indicating a pathological role for microglia [14, 41, 42]. In contrast, in our current experiments studying prion-induced PR degeneration, ablation of the microglia population using PLX5622 did not eliminate PR degeneration. Thus, microglia were not required for prion-induced retinal damage. In fact, the tempo of ONL thinning due to loss of PR cell nuclei was slightly faster in the absence of microglia (Fig. 4c and Fig. 5e). Therefore, microglia appeared to have an overall beneficial effect during prion retinal infection. Similar results were recently observed in an acute retinal detachment model where microglial elimination by PLX5622 did not block pathology, but instead, resulted in increased PR injury [29]. This pattern was also seen in prion-infected brain, where microglia ablation by PLX5622 led to earlier onset of pathology and reduced survival times [7].

The fact that microglia were not required for prion-induced retinal degeneration suggested that PrPSc generated in the retina from PrPC precursor molecules might initiate pathogenesis directly. After prion infection, PrPSc aggregates were noted first in the IS region at 82 dpi (Fig. 9d) and later also in the ONL (Fig. 9e-g, j). One to two weeks later the PR in these areas became disorganized ultimately disappearing almost entirely. This prion-induced retinal degeneration appeared to be quite cell-specific, involving photoreceptor neurons, but not other types of neurons or glia in the retina. The primary association of PrPSc with the IS region may be an important clue as to the pathogenic mechanisms involved. PrPC is expressed at the IS, and PrPSc appeared to be generated at this same location. Early localization of retinal damage to the IS area was previously seen in scrapie-infected hamsters [4]. The IS region is the site of most of the mitochondria of PR cells, and the IS is joined by the cilium to the outer segment of the PR cells containing the stacked discs of rhodopsin. Previous papers have suggested that normal PrPC might be colocalized to both mitochondria of brain cells [12, 13] and the ciliary basal body in neuroepithelial stem cells [16]. Generation of PrPSc at such PrPC sites in PR cells might alter redox energy metabolism by mitochondria or protein transport by the connecting cilia. Furthermore, proteasome overload by accumulation of misfolded proteins such as PrPSc may favor ciliopathy and PR degeneration as seen in other models [26, 31].

In the present paper, using transgenic mice with GFP and RFP under control of microglial or monocytic promoters respectively, we were able to distinguish the types of Iba1-positive cells associated with retinal degeneration in prion-infected mice with and without microglia. When microglia were ablated by PLX5622, there were considerably fewer Iba1-positive cells detected. Some of these cells appeared to be regenerating microglia, and other cells with a rounded shape and strong diffuse red fluorescence in cytoplasm were suggestive of RFP-positive monocytes, which might have been recruited from the periphery to remove the damaged PR cells. In contrast, in ND mice with microglia present, the main cells in degenerating retina were GFP-positive, RFP-negative, suggesting that they were microglia. Late in disease some cells were both green and red and might be dual-positive microglia. However, the red fluorescence in these cells was localized to distinct cytoplasmic vacuoles similar to phagolysosomes, suggesting that this red fluorescence might be due to phagocytosed outer segment material from PR cells, rather than RFP. We found similar cells in scrapie-infected mice which did not have an RFP-expressing transgene (Fig. 8c and d) and, others have noted similar autofluorescence of phagocytosed outer segment material in microglia and macrophages [25, 27]. In summary, at the end stage of retinal degeneration, Iba1-positive cells in ND mice were mostly microglia, whereas in PLX mice Iba1-positive cells were very reduced in number and were a limited mixture of monocytes plus regenerating microglia. Both cell types appeared to phagocytose damaged PR cells, but this occurred so late in the degenerative process that it did not appear to be a major causal mechanism of damage.

In previous experiments by our group, PLX5622-induced ablation of microglia was able to reduce survival times from scrapie prion infection by 21–32 days [7]. Thus, the presence of microglia in brain had an unexpected role in host defense against scrapie brain infection. In PLX5622-treated mice, PrPSc appeared in brain about 20 days earlier than in untreated mice suggesting that microglia might reduce PrPSc in brain by phagocytosis or other catabolic processes. In the present experiments studying retinal prion disease, microglial ablation by PLX5622 slightly accelerated prion-induced retinal degeneration. This effect might be due to the ability of microglia to catabolize PrPSc and reduce its damaging effects on retina as was suggested in previous brain experiments. However, the current retinal experiments were not able to detect differences between PLX-treated and ND mice in the amount of PrPSc in PR and ONL. More quantitative assays will be required to confirm this possibility. Moreover, it is possible that microglia can prolong retinal PR survival by other mechanisms, such as removing partially damaged PR cells to reduce bystander cell damage.

## Conclusions

The ablation of microglia with the use of PLX5622 in the present experiments revealed that microglia were not required for prion-induced PR degeneration. This conclusion was unexpected as there are many similarities between the pathology of prion-induced PR degeneration and genetic models of PR degeneration, such as rd10, where microglia are thought to play an active role in retinal damage [40–42]. However, the mechanism of prion-induced retinal pathogenesis might be a more direct toxic effect of PrPSc on PR cells, whereas rhodopsin mutants, such as rd10, might act indirectly via stimulation of neurotoxic effects induced in microglia. To distinguish between these mechanisms, PLX5622 ablation of microglia would be interesting to test in models of retinitis pigmentosa, AMD and other retinal degenerative conditions to explore the possibility that microglia might have different or additional roles than are currently appreciated.

## Abbreviations

GCL: Ganglion cell layer
INL: Inner nuclear layer
IPL: Inner plexiform layer
IS: Inner segment of photoreceptor cells
ONL: Outer nuclear layer, containing PR cell nuclei
OPL: Outer plexiform layer
OS: Outer segment of photoreceptor cells
PR: Photoreceptor(s)
RPE: Retinal pigment epithelium
